# Notes and Notices

**Published:** 1888-06

**Authors:** 


### NOTES AND NOTICES.

The I. H. A. will meet at Niagara, June 19–22d. Come one and all!

Removals.—Edward M. Gramm, to 1433 Girard Avenue, Philadelphia. Dr. W. L. Reed, from Mexico, Missouri, to 2309 Washington Avenue, St. Louis. Dr. Reed succeeds Dr. Kent. Dr. J. T. Kent, from St. Louis to 1419 Walnut Street, Philadelphia—a welcome addition to the force of Hahnemannians in Philadelphia.

Ohio Homœopaths met at Delaware May 8th and 9th; there was a good attendance. The officers elected were: Dr. C. E. Walton, President; Dr. C. L. Cleveland, First Vice-President; Dr. Frances J. Derby, Second Vice-President; Dr. F. Kraft, Secretary; Dr. C. D. Crank, Assistant Secretary; Dr. H. Pomeroy, Treasurer; Dr. H. E. Beebe, Chairman Board of Censors. The next meeting will be held at Cincinnati, second Tuesday in May, 1889.

New York Homœopathic Medical College held its annual commencement exercises in Chickering Hall, New York city, April 13th. A class of forty-eight was graduated. Addresses were made by the Dean, Professor T. F. Allen, Hon. Rufus B. Cowing, and Rev. Dr. Bowles. The prizes were awarded by Professor Malcolm Leal, the President of the faculty. The class valedictory was delivered by Dr. J. T. W. Kastendieck. Work on the new College building is to be begun at once, and pushed so as to be ready for use at the opening of the fall session of the College.

Lachesis, one of our most useful remedies, introduced and proven by Dr. Hering, has been given a very full and complete rendering in the forthcoming volume of the *Guiding Symptoms*. The editors give some ninety pages to this grand remedy, and not a line could be wisely omitted.

Clinical Work.—The Clinical Bureau of the I. H. A. for this session has promise of many good things. Dr. Alice B. Campbell, who has charge of its work, has been very active. Dr. Biegler will read papers on "Mental Alienation" and on Kali-phos. in a case of acute laryngitis; Dr Sawyer, "A Few Cases of Cancer;" Dr. Schmitt, "Clinical Cases Illustrating the Curative Power of Ant tart.;" Dr. Clark, "Some Symptoms of Sycosis;" Dr. Hawley, "A Case of Cancer of the Stomach Cured by Arsenicum."

Testimony for High Potencies.—Farrington wrote: "It is true of Natr-mur., as of most other drugs, that the high potencies act best." One of our subscribers recently wrote: "For the past three months I have been testing the higher attenuations or potencies, and am becoming highly interested in their use. My *prejudices* were *against* their efficacy, but I am *compelled* to admit that my practice during this period has been fully one hundred per cent. more satisfactory. R. B. W."

Mental Symptoms.—Hahnemann has always laid the greatest stress upon the importance of mental symptoms. They are to be covered in the prescription, as they are more indicative of the patient than are the pathological conditions, which are mainly indicative of the disease. In a foot-note (*Organon*, p. 187) Hahnemann illustrates the importance of mental symptoms by this remark: "Aconite seldom or never effects a rapid and permanent cure when the temper of the patient is quiet and even; nor Nux vomica when the disposition is mild and phlegmatic; nor Pulsatilla, when it is lively, serene, or obstinate; nor Ignatia, when the mind is unchangeable and little susceptible of either grief or fear."

Symptoms, Not Diseases.—*Trichiasis* is not a disease but a symptom, and as different as the occasions or causes are; as different as are the groups of symptoms it appears in connection with, and hence different remedies have to be indicated. *Borax* is one of the most important. If caused by the barbarous, ruthless, and nonsensical application of *Nitrate of Silver*, the *Natr-mur.* ought to be given first. *Graphites* may soften the scars as well as it does in the mammæ; if not, the callosities may require mechanical aid. Only *distichiasis* demands surgical treatment. *Entropium*, as well as *Ectropium*, in the beginning, and before it is spoiled by the surgeons, is in almost all cases cured by *Merc.* or *Sulph.*, sometimes *Calc.*, rarely *Lyc.*—C. Hering, in *Journal of M. M.*

